# Health workers’ compliance to rapid diagnostic tests (RDTs) to guide malaria treatment: a systematic review and meta-analysis

**DOI:** 10.1186/s12936-016-1218-5

**Published:** 2016-03-15

**Authors:** Alinune N. Kabaghe, Benjamin J. Visser, Rene Spijker, Kamija S. Phiri, Martin P. Grobusch, Michèle van Vugt

**Affiliations:** Public Health Department, College of Medicine, Private Bag 360, Blantyre, Malawi; Division of Internal Medicine, Department of Infectious Diseases, Center of Tropical Medicine and Travel Medicine, Academic Medical Center, University of Amsterdam, Meibergdreef 9, PO Box 22700, 1100 DE Amsterdam, The Netherlands; Centre de Recherches de Médicales de Lambaréné (CERMEL), Albert Schweitzer Hospital, Lambaréné, Gabon; Medical Library, Academic Medical Center, University of Amsterdam, Amsterdam, The Netherlands; Cochrane Netherlands, Julius Center for Health Sciences and Primary Care, University Medical Center Utrecht, Utrecht, The Netherlands

**Keywords:** Malaria, Rapid diagnostic test (RDT), Health workers, Sub-Saharan Africa, *Plasmodium falciparum*, Clinical decision making, Adherence, Compliance

## Abstract

**Background:**

The World Health Organization recommends malaria to be confirmed by either microscopy or a rapid diagnostic test (RDT) before treatment. The correct use of RDTs in resource-limited settings facilitates basing treatment onto a confirmed diagnosis; contributes to speeding up considering a correct alternative diagnosis, and prevents overprescription of anti-malarial drugs, reduces costs and avoids unnecessary exposure to adverse drug effects. This review aims to evaluate health workers’ compliance to RDT results and factors contributing to compliance.

**Methods:**

A PROSPERO-registered systematic review was conducted to evaluate health workers’ compliance to RDTs in sub-Saharan Africa, following Preferred Reporting Items for Systematic Reviews and Meta-Analyses (PRISMA) guidelines. Studies published up to November 2015 were searched without language restrictions in Medline/Ovid, Embase, Cochrane Central Register of Controlled Trials, Web of Science, LILACS, Biosis Previews and the African Index Medicus. The primary outcome was health workers treating patients according to the RDT results obtained.

**Results:**

The literature search identified 474 reports; 14 studies were eligible and included in the quantitative analysis. From the meta-analysis, health workers’ overall compliance in terms of initiating treatment or not in accordance with the respective RDT results was 83 % (95 % CI 80–86 %). Compliance to positive and negative results was 97 % (95 % CI 94–99 %) and 78 % (95 % CI 66–89 %), respectively. Community health workers had higher compliance rates to negative test results than clinicians. Patient expectations, work experience, scepticism of results, health workers’ cadres and perceived effectiveness of the test, influenced compliance.

**Conclusions:**

With regard to published data, compliance to RDT appears to be generally fair in sub-Saharan Africa; compliance to negative results will need to improve to prevent mismanagement of patients and overprescribing of anti-malarial drugs. Improving diagnostic capacity for other febrile illnesses and developing local evidence-based guidelines may help improve compliance and management of negative RDT results.

*Trial registration:* CRD42015016151 (PROSPERO)

**Electronic supplementary material:**

The online version of this article (doi:10.1186/s12936-016-1218-5) contains supplementary material, which is available to authorized users.

## Background

**Fig. 1:**
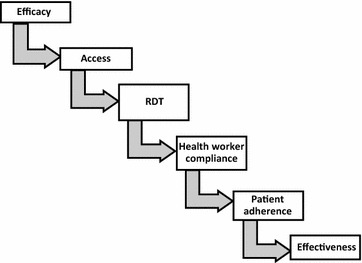
Pathway of health systems effectiveness of malaria diagnosis and treatment. (Adapted from MalERA consultative group) [6]

*Plasmodium falciparum* malaria is estimated to have caused 528,000 deaths and 163 million clinical episodes in sub-Saharan Africa in 2013 [1]. Early diagnosis and treatment with appropriate anti-malarial drugs can prevent severe illness and lethal outcome [2, 3]. Artemisinin-based combination therapy (ACT) is currently recommended for the treatment of uncomplicated malaria caused by *P. falciparum* [3, 4] and is increasingly used for non-falciparum malaria [5]. Effective case-management of malaria consists of an efficacious treatment, prompt access to treatment and diagnosis, provider compliance to treatment guidelines, and patient adherence to medication [3, 6] (Fig. 1).

Presumptive diagnosis and treatment of malaria based on symptoms leads to over- diagnosis of malaria and missed diagnosis for patients without malaria [7, 8]. The World Health Organization (WHO) recommends that any suspected malaria case in any epidemiological setting should be parasitologically-confirmed by either microscopy or rapid diagnostic test (RDT) before treatment [3]. Lack of trained personnel, equipment [9], and reagents for microscopy in most remote rural areas in Africa [10, 11] with high malaria burden makes the RDT the most practically suitable tool to confirm a malaria diagnosis [12, 13]. RDTs are immunochromatographic test kits which confirm the presence of malaria parasites in suspected patients by detecting one or a combination of the following three Plasmodium antigens: *Plasmodium* histidine-rich protein (HRP) 2 (pHRP-2) for *P. falciparum* or a ‘pan-specific’ aldolase to detect other species, such as *P. vivax* or *Plasmodium* lactate dehydrogenase (LDH) variants (pLDH) (with clonality specific to the various *Plasmodium* species infecting humans) [14, 15].

The use of malaria RDT can reduce over-prescribing of anti-malarial drugs (AMD). Studies have shown that in most endemic countries in sub-Saharan Africa, health workers of different cadres do not comply with malaria RDTs; they prescribe AMDs to patients with RDT negative results [13, 16–18]. This has implications on resources for patient, family members and health system since some drug combinations are relatively expensive [19–21]. Non-compliance to malaria negative results by prescribing AMDs neglects underlying cause of fever and expose patients unnecessarily to adverse effects; underlying infections, such as sepsis, pneumonia and meningitis [22–25], present as malaria clinically but are not routinely investigated [26] and may not be treated [10, 27].

To treat malaria effectively, to reduce costs and avoid unnecessary exposure to drug adverse effects, there is a need to correctly diagnose and comply with malaria treatment guidelines or clinical decision algorithms. Health workers (HWs) need to use the correct treatment based on the RDT results.

This systematic review examines data available on HWs compliance to RDT results in sub-Saharan Africa, and investigates factors associated with compliance to results (HW treating patients according to the RDT result). The primary outcome is the percentage of HWs compliant to overall, positive or negative, test results.

## Methods

This systematic review was registered in advance in the International prospective register of systematic reviews (PROSPERO; registration number CRD42015016151) which included pre-specified the objectives and inclusion criteria [28].

An experienced information specialist (RS) conducted a search without language or time restrictions in the online electronic databases Ovid Medline, Ovid Embase, Cochrane Central Register of Controlled Trials, CINAHL Plus with Full Text, African Index Medicus, and African Journals Online (AJOL). The search used both free text words and medical subject headings for ‘malaria’, ‘RDT’, ‘health worker’ and ‘compliance’. The search was conducted on 3 March 2015 and updated on 12 November 2015. Studies reporting on malaria suspected patients of any age presenting to HWs of any cadre in sub-Saharan Africa were searched. The intervention was the use of a WHO recommended RDT kit for parasitological confirmation of a malaria diagnosis (a list of WHO recommended RDTs is available online [29]).

Bibliographies of relevant studies retrieved from the studies were checked for additional publications. The search strategy is described in Additional file 1. EndNote X7.4 (Thomson Reuters) was used to manage, de-duplicate and screen the references for eligibility. The inclusion criteria were: studies were conducted in sub-Saharan Africa; RDTs were used to diagnose malaria in symptomatic patients; the RDTs used were WHO-recommended; absolute numbers of RDT result adherence as primary or secondary outcome were reported. Exclusion criteria were: studies using RDT for active case finding and population screening; conference abstracts; no absolute numbers were reported; studies outside sub-Saharan Africa. Eligibility assessment of studies was performed independently in a blinded, standardized way by two reviewers (ANK and BJV). Titles and abstracts were screened first, and the two reviewers screened and selected relevant full-text articles. ANK extracted quantitative data based on the pre-specified criteria into an excel sheet (Additional file 2); factors associated with compliance were also extracted into the same sheet. All the quantitative data was independently checked by BJV. Data extracted included author name, year of publication, place of study, transmission setting, type of RDT, cadre and number of HW, age of patients, number of test results, RDT positives treated and RDT negatives not treated. Both qualitative and quantitative factors were also extracted from included studies which reported them. The risk of bias of studies was not assessed because of the diversity of the study designs included.

**Fig. 2:**
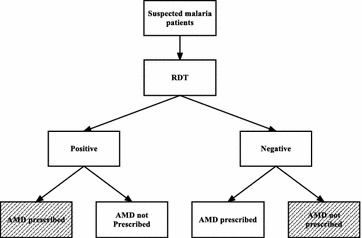
Patient pathway for malaria diagnosis and treatment. The *shaded areas* represent appropriate management (*RDT* rapid diagnostic test, *AMD* anti-malarial drug)

The primary outcome measure was proportions in percentage of RDT results with appropriate AMD prescription disaggregated to positive and negative results adherence. Appropriate treatment was defined as AMDs prescribed to RDT positive and AMD not prescribed to RDT negative patients (Fig. 2). Formulae for these calculations are included in Additional file 3. STATA version 13 (StataCorp, College Station, TX, USA) was used to calculate the pooled estimate of proportions appropriately treated overall and negative and positive compliance using random effects. Random effects analysis was used after an initial fixed effect analysis had I^2^ above 50 %, suggesting heterogeneity. Pooled estimates were also stratified by health personnel cadre, age of patients and malaria transmission setting. A qualitative synthesis of factors contributing to compliance was also reported for the included studies.

## Results

### Study selection

**Fig. 3:**
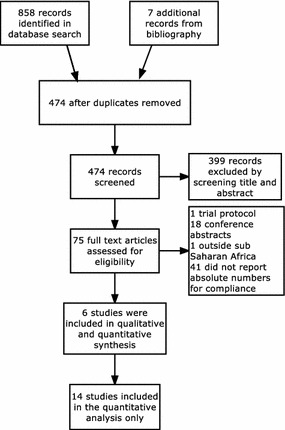
Study selection flow (PRISMA)

The total number of articles after removing duplicates was 474 (Fig. 3). After screening title and abstracts for eligibility, 75 full-text articles were examined for eligibility; 14 studies were included in the quantitative analysis [7, 16–18, 30–39]. Five of the studies reported on factors associated with compliance to RDT results and were included in the summary of associated factors [7, 17, 30, 31, 36].

There were five study designs (Table 1): one randomized control trial [31], four observational [32–34, 38], four cross-sectional [17, 18, 36, 37], four cluster randomized trials [7, 16, 30, 35] and pre-post intervention study [39]. RDT adherence was a secondary outcome in five out of the 14 studies [17, 31, 35, 37, 39].

**Table 1:** Characteristics of studies included

Authors	Year	Country	Study setting	Study design	HW cadre	Number of HWs	Age of study participants	RDT	Sample size
Bisoffi [31]	2009	Burkina Faso	Stable malaria with seasonal transmission	RCT	Nurses	NR	>6 months	Paracheck Pf	1050
Masanja [33]	2010	Tanzania	Holoendemic	Observational	Clinicians	99	>5 years	ParaHIT	10,650
Bottieau [32]	2013	Mozambique	Perennial transmission with seasonal peaks	Observational	Clinicians	NR	All	Paracheck Pf; ICT malaria Pf; SD Bioline Pf	1385
Manyando [38]	2014	Zambia	Both low and high transmission	Observational	Clinicians	NR	<5 years	ICT malaria Pf	1492
Chinkhumba [37]	2010	Malawi	Stable malaria with seasonal peak	Cross sectional	Clinicians and nurses	NR	>5 years	ICT malaria pf; SD Bioline; Paracheck Pf; First Response	1390
Uzochukwu [36]	2011	Nigeria	High transmission	Cross sectional	Clinicians, nurses and CHW	32	All	ICT malaria Pf	280
Mubi [17]	2013	Tanzania	Perennial transmission	Cross sectional	Clinicians and nurses	20	>3 months	NR	105
Shakely [18]	2013	Zanzibar	Low transmission	Cross sectional	Clinicians and nurses	33	All	Paracheck Pf	3889
Batwala [30]	2011	Uganda	Both low and high transmission	CRT	Clinical officers and nurses	30	All	Paracheck Pf	44,565
Mukanga [35]	2012	Ghana, Uganda	Seasonal	CRT	CHW	44	4–59 months	Paracheck Pf; ICT malaria Pf	1559
Mbacham [16]	2014	Cameroon	NR	CRT	Clinicians	198	All	SD Bioline	1194
Bastiaens [39]	2011	Tanzania	NR	Before and after	Clinical officers	NR	Below 10 year olds	ICT malaria Pf; Paracheck Pf	501
Mbonye [7]	2015	Uganda	Perennial transmission	CRT	DSV	10	All	First response	8073
Mukanga [34]	2011	Uganda	High transmission	Observational	CHW	14	Under 5 years	NR	182

*CHW* community health worker, *CRT* cluster randomized trial, *DSV* drug shop vendor, *NR* Not reported, *RCT* randomized control trial

### Health workers’ compliance to malaria results

**Fig. 4:**
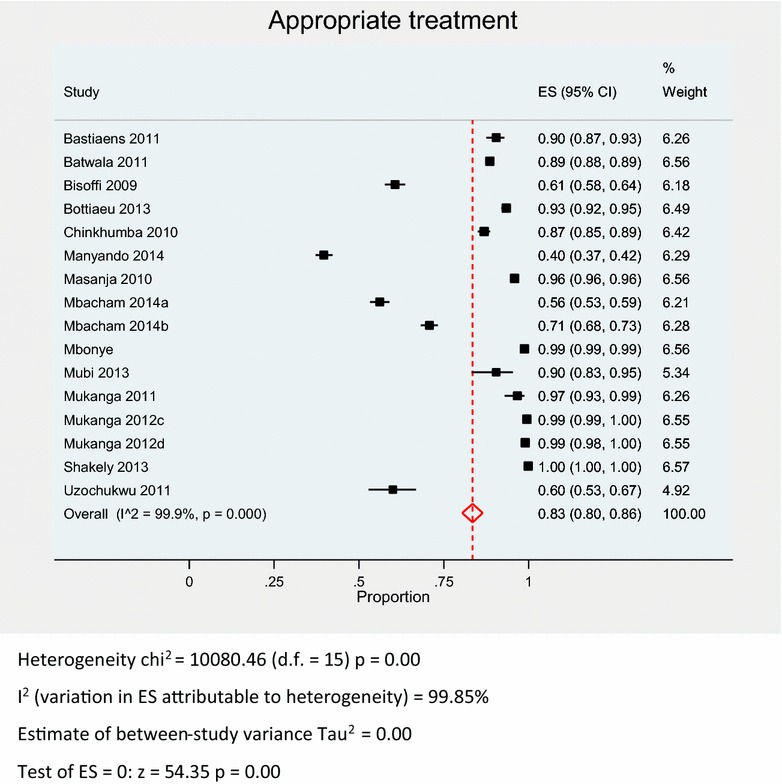
Pooled meta-analysis of overall compliance to RDT results

**Fig. 5:**
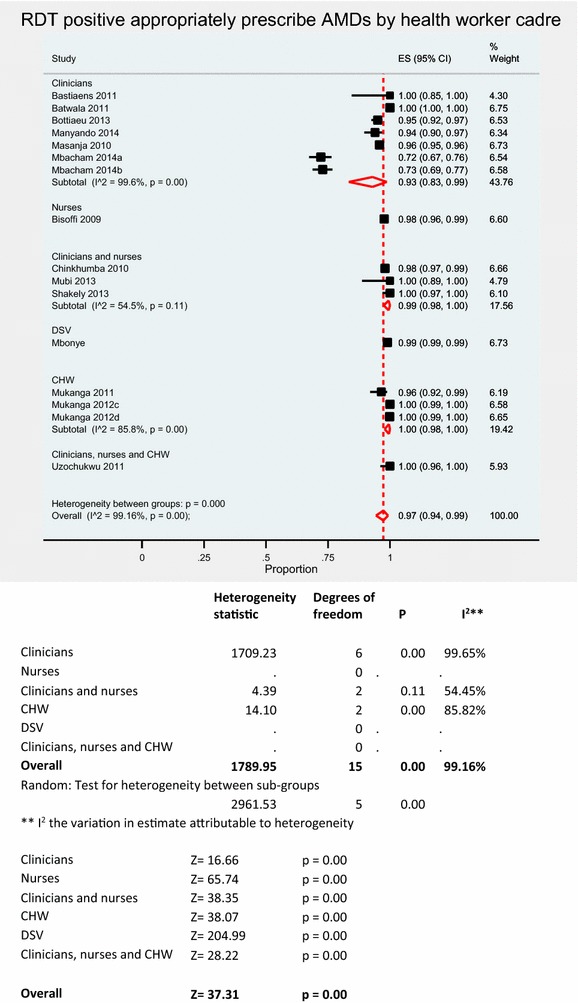
Pooled meta-analysis of RDT positive results appropriately prescribed AMDs stratified by HW cadre

A pooled meta-analysis using random effects (Fig. 4) for the 14 studies [7, 16–18, 30, 31, 33–40] shows an overall compliance of 83 % (95 % CI 80–86 %); I^2^ = 99.9 %, Z = 54.35, p < 0.001. Appropriate malaria treatment based on RDT results (Table 2) was as low as 39.7 % in a Zambian study [38] to as high as 99.9 % in Zanzibar [18]. The pooled meta-analysis result using random effects for RDT positives prescribed AMDs (Fig. 5) was 97 % (95 % CI 94–99 %); I^2^ = 99.2 %, Z = 37.31, p < 0.001. The proportion of positive RDT results prescribed AMDs ranged from 72.1 to 100 %. 12 studies reported appropriate prescription of AMDs to RDT positive patients above 93 %; six of these studies had 100 % RDT positive compliance (Table 2).

**Table 2:** Appropriate treatment overall, RDT positive and RDT negative results

Study design	Authors	Country	Health personnel cadre	Appropriate treatment (%)	Positives treated (%)	Negatives not treated (%)
RCT	Bisoffi	Burkina Faso	Nurses	60.7	97.7	19.0
Observational	Masanja	Tanzania	Clinicians	95.9	95.8	96.0
	Bottiaeu^a^	Mozambique	Clinicians	93.4	95.1	92.8
	Mukanga	Uganda	CHW	97.8	98.6	95.2
	Manyando	Zambia	Clinicians	39.7	93.9	31.4
Cross sectional	Chinkhumba	Malawi	Clinicians and nurses	86.9	98.0	57.9
	Uzochukwu	Nigeria	Clinicians, nurses and CHW	60.0	100.0	25.9
	Mubi	Tanzania	Clinicians and nurses	90.5	100.0	86.5
	Shakely	Zanzibar	Clinicians and nurses	99.9	100.0	99.9
CRT	Batwala	Uganda	Clinical officers and nurses	88.5	100.0	76.6
	Mukanga^b^	Ghana	CHW	99.5	100.0	96.7
	Mukanga^b^	Uganda	CHW	99.0	99.9	92.4
	Mbacham^c^	Cameroon	Clinicians	56.1	72.1	48.1
	Mbacham^d^	Cameroon	Clinicians	70.8	72.9	69.4
	Mbonye	Uganda	DSV	98.8	99.0	98.5
Before and after	Bastiaens	Tanzania	Clinical officers	90.4	100.0	90.0

*CHW* community health worker, *DSV* drug shop vendors

^a^ Excludes missing data

^b^ Excludes Burkina Faso results

^c^ Basic training

^d^ Enhanced training

**Fig. 6:**
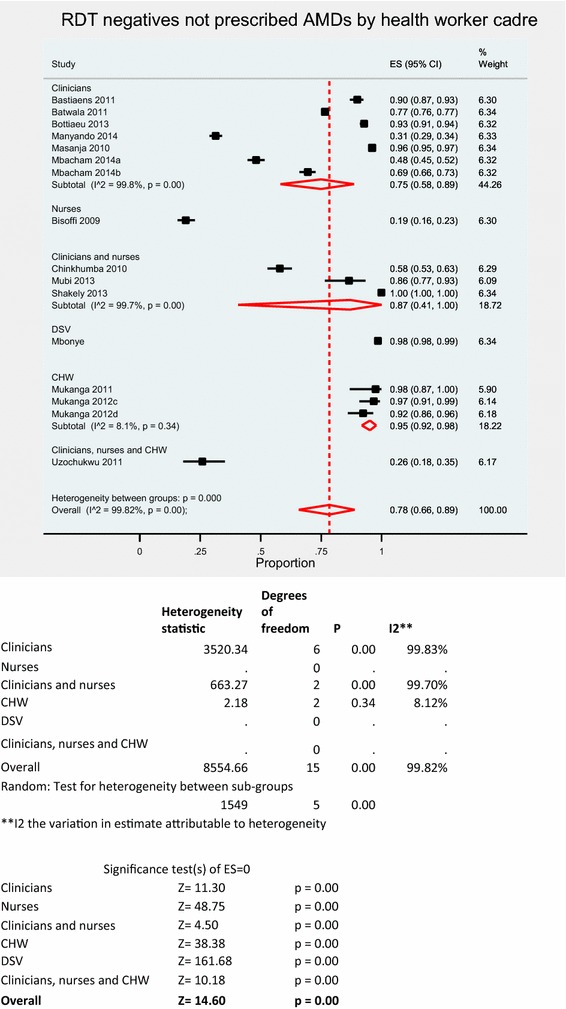
RDT negative results not prescribed AMD stratified by HW

Pooled meta-analysis using random effects for RDT negative patients not prescribed AMDs (Fig. 6) was 78 % (95 % CI 66–89 %); I^2^ = 99.8 %, Z = 14.60, p < 0.001. The proportion of RDT negative patients appropriately not prescribed and AMD was between 19.0–99.9 % (Table 2). Five studies [16, 31, 36–38] reported less than 60 % compliance to RDT negative results.

Community health workers (CHWs) had the highest adherence to negative results (Fig. 6) with a random effects pooled proportion of 95 % (95 % CI 92–98 %); I^2^ = 86.4 %, Z = 23.26, p < 0.001 than clinicians with a pooled proportion of 75 % (95 % CI 58–89 %); I^2^ = 99.8 %, Z = 11.30, p < 0.001. There were no differences in compliance when stratified by patient age or transmission setting in pooled meta-analyses.

### Compliance factors

Six out of the 14 studies included [7, 17, 30, 31, 36, 38] reported quantitative or qualitative assessment of factors associated with compliance to RDT. Uzochokwu et al. [36] reported that HWs adhered to RDT positive results, as they believed they were more reliable in confirming a malaria diagnosis than presumptive diagnosis or microscopy. Bisoffi et al. [31] compared prescribing behaviour of HWs in the dry compared to rainy seasons and reported improved RDT negative results compliance during the dry season; alternative diagnoses were also made in the dry than the rainy season.

Manyando et al. [38] reported no association between prescribing of AMD to negative RDTs in children under five, and fever in a Zambian study. There was also no association between community health worker (CHW) or socio-demographic characteristics and classification of malaria based on RDT in a bivariate analysis in Uganda [34]. In one study though, 70 % (14/20) of the respondents (HW) believed that RDTs gave inaccurate/false negative results for malaria [17]. Persistence of symptoms and patient pressure and demand were other factors reported to contribute to inappropriate AMD prescription in RDT negative cases [7, 17, 41]. HWs would end up prescribing AMDs in these cases to satisfy patients and maintain their reputation. Some HW reported that RDT negative patients improved when they were prescribed AMDs.

## Discussion

This is the first systematic review and meta-analysis evaluating the proportion of health workers’ compliance with RDT results. Overall the compliance is fair. However, it also confirms that compliance to RDT negative results compared to positive results was generally low among HWs.

Diagnostic accuracy of RDT for both falciparum and non-falciparum malaria is high [14, 15]; sensitivity of up to 99.5 % and specificity of up to 90.6 % compared to microscopy for *P. falciparum* [14]. Community health workers can appropriately diagnose and treat malaria using RDT in resource limited settings [13]. The use of RDT to guide treatment reduces AMD prescription especially where health workers adhere to results [42].

The results show a high proportion of HWs prescribe appropriate treatment based on RDT results. A proportion of patients still remain over- or under-treated, despite policy change of administering ACT to parasitological confirmed cases only. Approximately 17 % of RDT negative patients are inappropriately prescribed AMDs. This estimate, extrapolated to sub-Saharan Africa means hundreds of thousands of patients are inappropriately diagnosed for malaria and prescribed AMD drugs unnecessarily; unnecessary (=incorrect) AMD prescription leads to drug wastage, unnecessary exposure to drug adverse effects and an increased risk of drug resistance development for current AMDs [43]. Where underlying infection is not treated, the patient’s illness prolongs and worsens; the patient or guardian makes multiple visits to seek health services, lose productivity time or income and leads to school absenteeism for school-going children [21] leading to a vicious cycle of poverty and malaria [44].

Lower cadres of HW showed more compliance to RDT results than trained HWs. The high adherence is likely due to trust in RDT result for confirming malaria diagnosis. Trained HWs on the other hand may trust clinical symptoms and past experience more than RDT result [17, 45].

Factors associated with HW compliance from qualitative studies include knowledge of alternative diagnosis, fever during the dry season and a trust in RDT result [30, 31, 46]. Trust may be increased by improving diagnostic capacity for other common febrile illnesses, and by developing evidence informed guidelines for treatment of symptomatic RDT negative patients. Such guidelines may not apply in non-endemic areas and therefore should be specific to particular settings.

Knowledge of alternative diagnosis is related to the level of training and experience of HW [47]. HWs reported they likely made alternative diagnosis during the dry season when malaria transmission is perceived lower in febrile children with negative RDT result compared to the wet season when transmission peaks. For febrile patients, alternative diagnoses were made during the dry season while more patients were treated for malaria during the wet season in one study [31].

Qualitative studies report pressure on prescribers to satisfy patient expectations as one factor, which contributes to non-compliance of RDT negative results [44, 48]. Chandler et al. [49] reported patient psychology and prescriber reputation as other factors influencing non-compliance to of HWs to negative RDT.

Interventions to improve compliance have not been successful, although they led to a decrease in ACT prescriptions in particular. Some HWs prescribed a non-recommended AMD in malaria negative patients [16, 50].

In cases of patients demanding AMDs, community sensitisation on RDTs was reported to improve patient satisfaction [7]. At facility level, involvement of patient in discussing malaria results also improved patient satisfaction and reduced patient demand for AMDs [51].

Notably, few studies were available which quantified HW’s compliance to malaria RDT results, and even less studies investigated the factors contributing to compliance. Understanding these factors can help design effective strategies to improve compliance of anti-malarial drugs. Chandler et al. [49] describe a systematic method of designing an intervention in Tanzania; formative research would be key in designing such an intervention. However, interventions are context-specific and may not be applicable to all settings, and for all HWs. It is essential to investigate factors contributing to non-compliance in specific cadres and settings, exploring impact in a context specific manner before designing and implementing interventions.

Although ideal for rural areas in Africa, RDT kits inherently are not 100 % sensitive and specific [14, 42]. Clinically diagnosed malaria and positive malaria test may be due to other underlying causes of the fever [27]. Crump et al. reported only 1.6 % of 820 patients with fever or history of fever actually had malaria infection in a Tanzanian prospective cohort study; bacterial and fungal bloodstream infections were responsible for 9.8 and 2.9 % of the fever, respectively. Resource limited settings lack diagnostic equipment and capacity for some diseases. Diagnostic accuracy of RDTs can be affected further by low and extremely high parasite densities [52, 53], patient-intrinsic factors such as rheumatoid factor positivity [54], user factors such as result interpretation and performance of the test, and environmental storage conditions including high temperatures. It is, therefore, possible, though infrequent, for malaria-infected patients to have a false positive (leading to not-indicated treatment) or more importantly, false negative result, and hence miss malaria treatment if WHO malaria treatment guidelines are followed. A more robust and highly specific test may be useful to rule out malaria. False positives, where malaria parasitaemia is not the cause of the illness (in endemic areas) lead to neglecting of other febrile illnesses.

Multidisciplinary research to explore, measure and design interventions for increasing compliance to RDT results in different settings in Africa need to be conducted. More innovation in diagnosis of common febrile illnesses in malaria endemic regions needs to be available. There is sparse data on prevalence of other non-malaria febrile illnesses in most malaria endemic regions of Africa.

The meta-analysis may have overestimated compliance: studies evaluating diagnostic tests generally report higher compliance when assessed in the study setting compared to a non-study setting. Most studies reported higher compliance to positive results compared to negative results.

A limitation for the results in the review is that risk of bias and publication bias were not assessed for the studies included; the quality of evidence therefore cannot be reported.

## Conclusion

HWs compliance to RDT is fair; compliance to positive RDT results is generally higher compared to negative RDT results. Over-treatment of malaria is still a major problem in sub-Saharan Africa. Both HW and patient factors contribute to inappropriate prescribing of AMDs to RDT negative patients; interventions to improve compliance should target both patients and HWs. Treatment guidelines should be developed for other causes of fever informed by local context and research. Multidisciplinary research will improve compliance of HWs to RDT results.

## Additional files

10.1186/s12936-016-1218-5 Search strategy.
10.1186/s12936-016-1218-5 Data extraction form.
10.1186/s12936-016-1218-5 Formulae for appropriate treatment.

## Abbreviations

ACT: artemisinin-based combination therapy
AL: artemether-lumefantrine
AMC: Academic Medical Center
AMDs: anti-malarial drugs
CERMEL: Centre de Recherches Médicales de Lambaréné
CI: confidence interval
CHW: community health worker
HW: health worker
RDT: rapid diagnostic test
WHO: World Health Organization
