# Supplementary material for: Health workers’ compliance to rapid diagnostic tests (RDTs) to guide malaria treatment: a systematic review and meta-analysis
Source: Malar J. 2016 Mar 15;15:163. doi: 10.1186/s12936-016-1218-5 (PMC4791859; doi:10.1186/s12936-016-1218-5)
Supplement: Supplementary file 3 — 10.1186/s12936-016-1218-5 Formulae for appropriate treatment. [file 12936_2016_1218_MOESM3_ESM.docx]

**Additional file 3: Formulae for appropriate management**

| Appropriate treatment= | RDT positive prescribed AMD + RDT negative not prescribed AMD |
| --- | --- |
|  | Total tested |

| Positive compliance= | RDT positive prescribed AMD |
| --- | --- |
|  | Total tested positive |

| Negative compliance= | RDT negative not prescribed AMD |
| --- | --- |
|  | Total tested negative |
